# Wearable sensors based on artificial intelligence models for human activity recognition

**DOI:** 10.3389/frai.2024.1424190

**Published:** 2024-06-27

**Authors:** Mohammed Alarfaj, Azzam Al Madini, Ahmed Alsafran, Mohammed Farag, Slim Chtourou, Ahmed Afifi, Ayaz Ahmad, Osama Al Rubayyi, Ali Al Harbi, Mustafa Al Thunaian

**Affiliations:** ^1^Department of Electrical Engineering, College of Engineering, King Faisal University, Al-Ahsa, Saudi Arabia; ^2^Department of Computer Science, College of Computer Science and Information Technology, King Faisal University, Al-Ahsa, Saudi Arabia

**Keywords:** human body motion, inertial measurement unit, barometer, fall detection, machine learning, convolutional neural network, sensors, sensor networks

## Abstract

Human motion detection technology holds significant potential in medicine, health care, and physical exercise. This study introduces a novel approach to human activity recognition (HAR) using convolutional neural networks (CNNs) designed for individual sensor types to enhance the accuracy and address the challenge of diverse data shapes from accelerometers, gyroscopes, and barometers. Specific CNN models are constructed for each sensor type, enabling them to capture the characteristics of their respective sensors. These adapted CNNs are designed to effectively process varying data shapes and sensor-specific characteristics to accurately classify a wide range of human activities. The late-fusion technique is employed to combine predictions from various models to obtain comprehensive estimates of human activity. The proposed CNN-based approach is compared to a standard support vector machine (SVM) classifier using the one-*vs*-rest methodology. The late-fusion CNN model showed significantly improved performance, with validation and final test accuracies of 99.35 and 94.83% compared to the conventional SVM classifier at 87.07 and 83.10%, respectively. These findings provide strong evidence that combining multiple sensors and a barometer and utilizing an additional filter algorithm greatly improves the accuracy of identifying different human movement patterns.

## 1 Introduction

**Figure 1:**
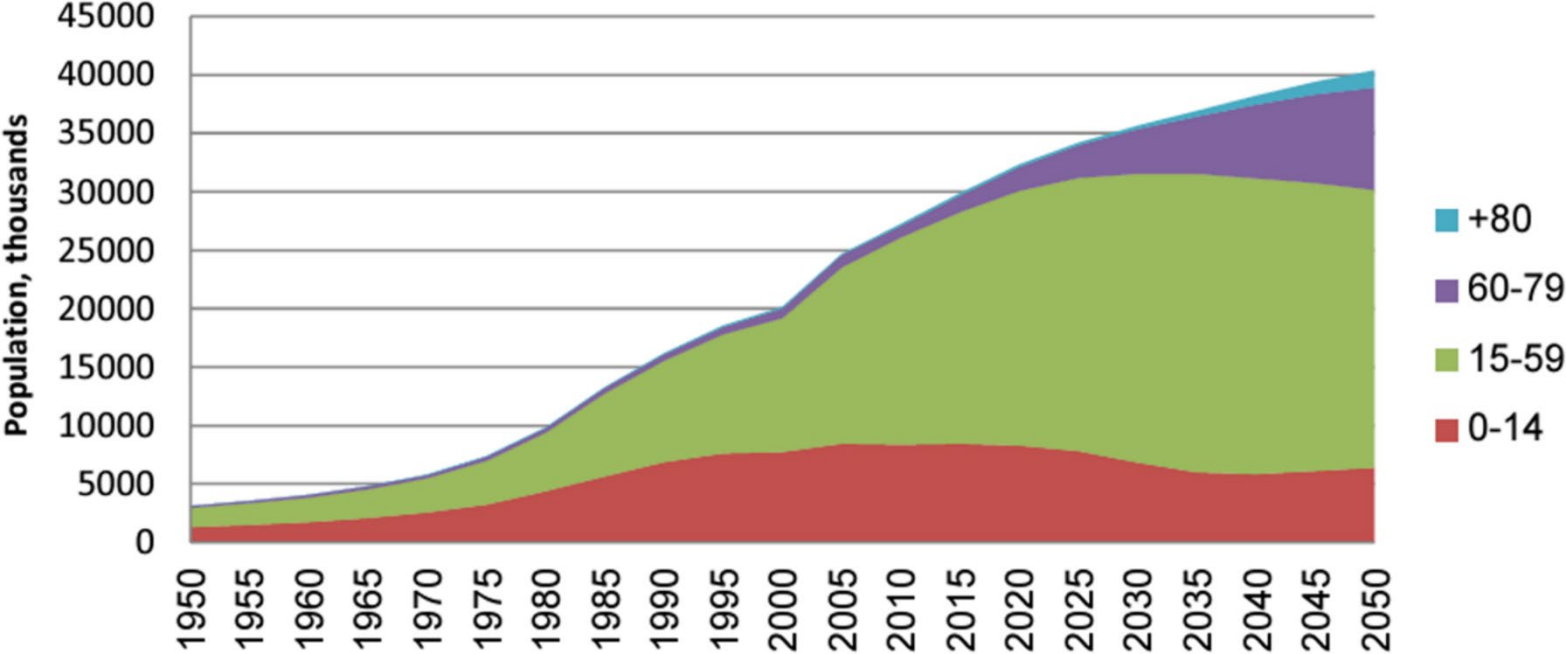
The Saudi Arabian population by age group is in the thousands (Abusaaq, 2015).

The elderly demographic is rapidly expanding and is expected to accelerate significantly in the 21st century. This projection is based on an analysis conducted by the United Nations (UN) examining global population aging trends from 1950 to 2050. Based on the UN, the population of Saudi Arabia will increase to 40 million by 2050, with a quarter of this population (i.e., 10 million individuals) aged 60 years or older. The population’s age distribution in Saudi Arabia during the period 1950–2050 is depicted in Figure 1.

**Figure 2:**
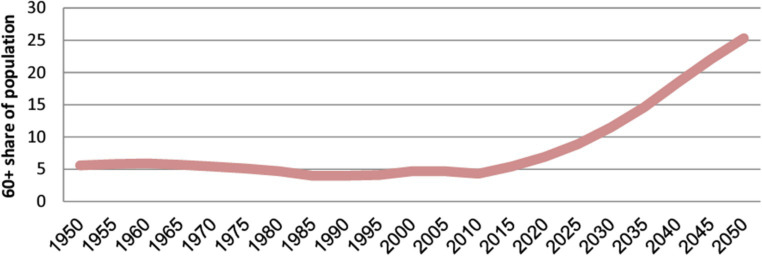
Share of the population aged over 60 years in Saudi Arabia (Abusaaq, 2015).

Cohorts aged 60–79 years and those aged above 80 years are currently experiencing particularly pronounced growth. In addition, there has been a consistent increase of approximately 5% in the number of individuals aged 60 years and over from 1950 to 2015, as illustrated in Figure 2.

Human activity recognition (HAR), a research hotspot in academia and industry aiming to further ubiquitous computing and human–computer interactions, is utilized in healthcare, fitness, gaming, tactical military operations, and indoor navigation. Wearable sensors and external equipment (e.g., cameras and wireless RF modules) represent two basic HAR systems. In sensor-based HAR, sensors are worn on the body to capture segmented and precise sensor signal patterns (Alarfaj et al., 2021).

There are many proposed machine learning (ML) algorithms for HAR prediction, with the five main types of algorithms as follows: algorithms based on fuzzy logic (FL) (Medjahed et al., 2009; Schneider and Banerjee, 2021), algorithms based on probabilities (Maswadi et al., 2021; Schneider and Banerjee, 2021), algorithms based on rules (Hartmann et al., 2022; Radhika et al., 2022), algorithms based on distance (Agac et al., 2021; Fahad and Tahir, 2021), and optimization-based approaches (Muralidharan et al., 2021; Nguyen et al., 2021). The six actions recognized in HAR, including exercise, lying down, sitting, standing up, walking, and sleeping, are recognized by fuzzy rule-based inference systems using FL (Medjahed et al., 2009). Recently, a new method for HAR using first-person video and fuzzy rules for inference was reported (Schneider and Banerjee, 2021).

This study presents a novel methodology for enhancing HAR using sensor-specific convolutional neural networks (CNNs). Each CNN is designed to the unique data characteristics and shape of a particular sensor type (accelerometer, gyroscope, or barometer), facilitating effective processing and accurate classification of a wide range of human activities. The methodology incorporates a late-fusion technique to integrate predictions from these diverse models, generating a comprehensive and accurate estimation of human activity. This approach addresses the limitations of single-model methods, using the strengths of individual sensor-specific CNNs for improved performance.

The novelty of this study lies in developing the sensor-specific CNN architecture, which enables the effective capture and utilization of distinctive features inherent to each sensor type, enhancing activity classification accuracy. This research overcomes the constraints of single-model approaches by implementing the late-fusion technique, which aggregates predictions from individual CNNs to comprehensively and accurately estimate human activity.

This study significantly contributes to the field of HAR by demonstrating the superior performance of the proposed late-fusion CNN model compared to the traditional support vector machine (SVM) classifier. This model’s enhanced accuracy and robustness can revolutionize healthcare applications, enabling advanced monitoring, early detection of health issues, and personalized interventions for improved patient outcomes.

## 2 Study background

The increased utilization of wearable sensors has stimulated notable progress in HAR. Although early-fusion approaches have been prominent in industry, late-fusion methods are becoming more popular because of their potential for modularity, interpretability, and enhanced performance in specific situations. This section examines prominent late-fusion techniques for HAR and contrasts them with the CNN-based late-fusion method developed in this study. Many studies have investigated late-fusion methods for HAR, employing various sensor modalities and fusion algorithms. Hammerling and Rosipal (2013) used late fusion with support vector machines (SVMs) on accelerometer and gyroscope data for classification HAR, which resulted in good accuracy but limited interpretability. Yang et al. (2017) introduced a majority voting method for late fusion, which revealed promising outcomes but can have neglected intricate interconnections among different modalities. Wang et al. (2016) employed a layered generalization model to integrate data from an accelerometer, a gyroscope, and a barometer. Although this approach yielded better results than utilizing each model individually, more processing resources were required. Zhang et al. (2019) combined data from various modalities before inputting them into a deep neural network, resulting in high accuracy. However, this approach can have overlooked inter-modal relationships. Sun et al. (2018) introduced a hybrid method integrating early- and late-fusion techniques with deep learning (DL) models. This strategy demonstrated better results than fusion strategies; however, the fusion architecture must be meticulously designed. Yu et al. (2020) employed early fusion to extract features and late fusion for decision-making using a deep neural network. Although the model showed good accuracy and robustness, its complexity increased. The CNN-based late-fusion approach proposed in this study presents numerous advantages compared to previous research. Utilizing separate CNNs for each sensor modality enables customized extraction of features specific to each data type to capture more comprehensive and distinguishing information than generic techniques that fuse features at a higher level. The study utilizes a late-fusion technique where the predictions from separate CNN models for each sensor (accelerometer, gyroscope, and barometer) are combined at the decision level. Each CNN model processes its sensor input independently and generates predictions for human activity. The individual predictions are aggregated through a weighted average or voting mechanism to get the final prediction.

The widespread adoption and advancement of neural networks have led to the displacement of conventional methods by DL techniques in solving HAR problems. Many studies have employed CNNs to perform activity categorization tasks using sensor data (Moya Rueda et al., 2018; Demrozi et al., 2020; Mahmud et al., 2021; Sikder et al., 2021). In addition, Sikder et al. (2021) evaluated the effectiveness of one-dimensional and two-dimensional (2D) sequential CNN models for classifying HAR signals. The results indicated that 2D CNNs yield superior results and surpass traditionally created models. The DL models were developed to classify HAR tasks. Xu et al. (2019) introduced the InnoHAR model, which combines an inception neural network with a recurrent neural network. iSPLInception drew inspiration from Google’s Inception-ResNet architecture and delivered superior predicted accuracy with reduced device resource requirements for signal-based HAR (Ronald et al., 2021). Hybrid models incorporating long short-term memory (LSTM) and bi-directional LSTM have become increasingly popular in recent studies for human activity classification as they are adept at extracting spatial and temporal properties (Hayat et al., 2022; Khan et al., 2022; Li and Wang, 2022; Luwe et al., 2022). Zhao et al. (2022) used a hierarchical LSTM CNN to classify farmers’ behavior in agriculture. Zhang et al. (2017) addressed gesture recognition by employing two types of neural networks: 3DCNN and ConvLSTM. In addition, many studies have used DL and ML to predict HAR (Almabdy and Elrefaei, 2019; Xu et al., 2019; Mutegeki and Han, 2020; Zheng et al., 2021).

## 3 Materials and methods

**Figure 3:**
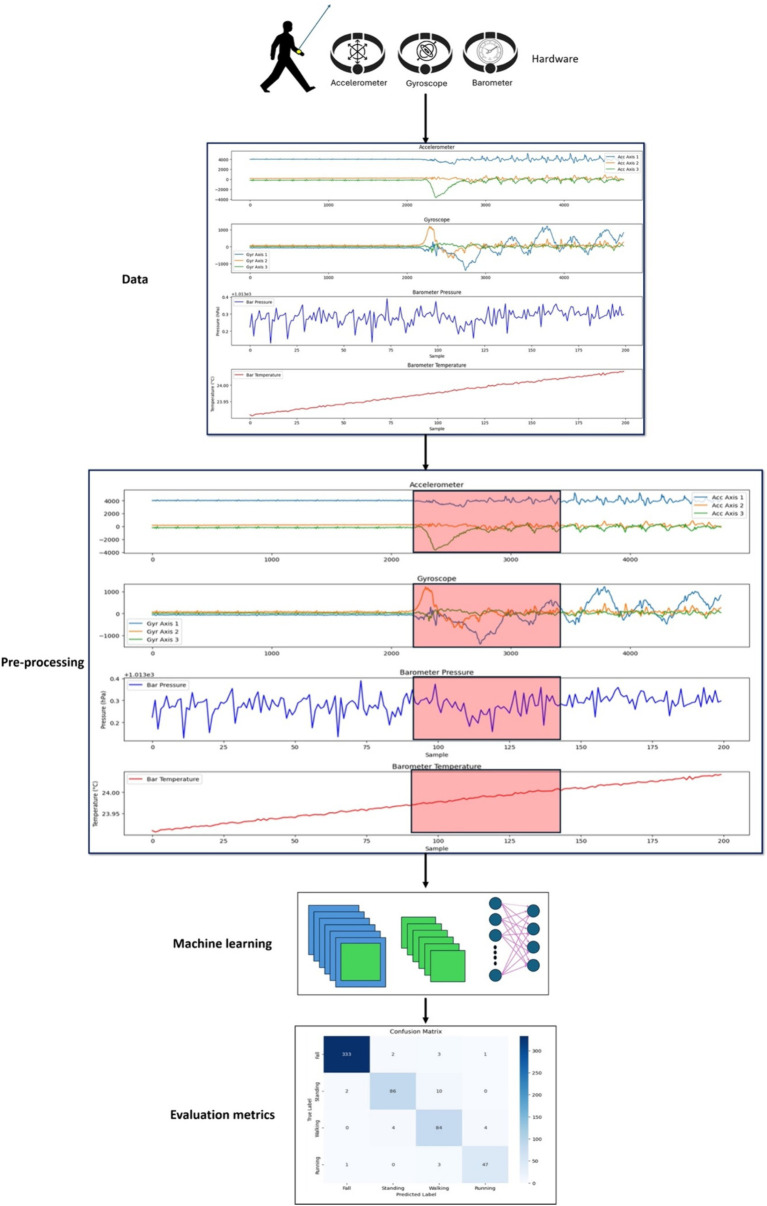
Framework of the HAR system.

The primary objective of this study is to develop a continuous human movement monitoring system capable of acquiring user movement data and accurately and efficiently transmitting them to a remote server. A wearable device in the form of a bracelet is designed to serve three primary functions: monitoring human body movement, fall detection, and localization. In addition, the bracelet can measure heart rate, pulse oximetry, and body temperature. In addition, an alarm system is integrated to become activated in response to concerns regarding declines in the user’s vital signs. This bracelet-type wearable device is selected for several compelling reasons: first, its accuracy remains unaffected by external factors such as weather, location, and time; second, the utilization of compact electronic components contributes to its low-power consumption; and third, it bears extensive adaptability, including minimal distance limitations, the capability to process and analyze substantial volumes of data, and user-friendly portability. Figure 3 displays the proposed framework of the HAR.

### 3.1 Hardware

**Figure 4:**
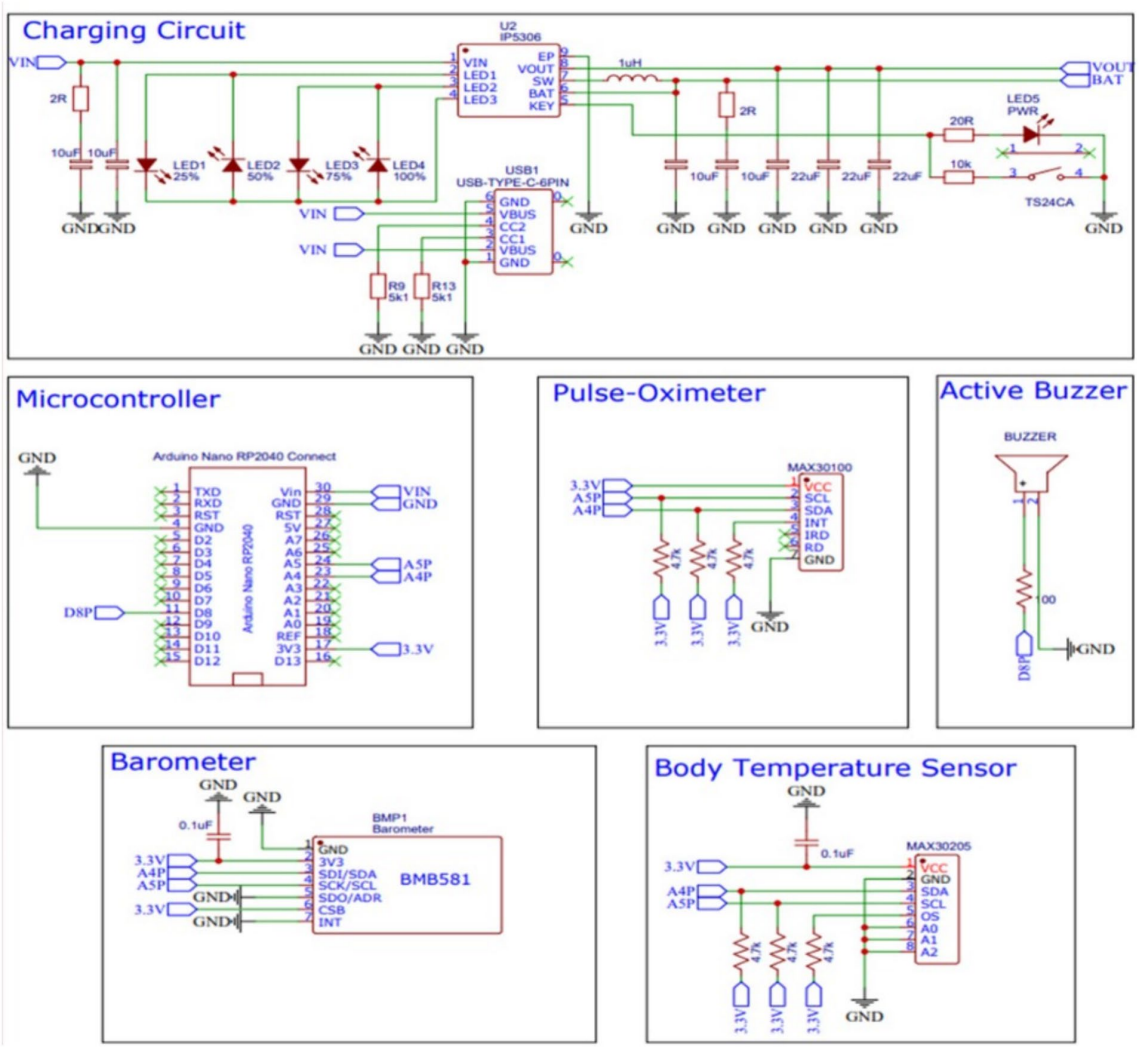
Hardware block diagram.

As the detection of movement patterns can be enhanced by combining multiple sensors, this study employs five distinct sensors: an inertial measurement unit (IMU), a barometer, a human body temperature sensor, a pulse-oximeter sensor, and an active buzzer. Each sensor is assigned a specific role with the goal of increasing the accuracy and precision of pattern detection. All these sensors are interconnected with a single microcontroller. The hardware block diagram is depicted in Figure 4.

#### 3.1.1 Arduino Nano RP2040 connect

**Figure 5:**
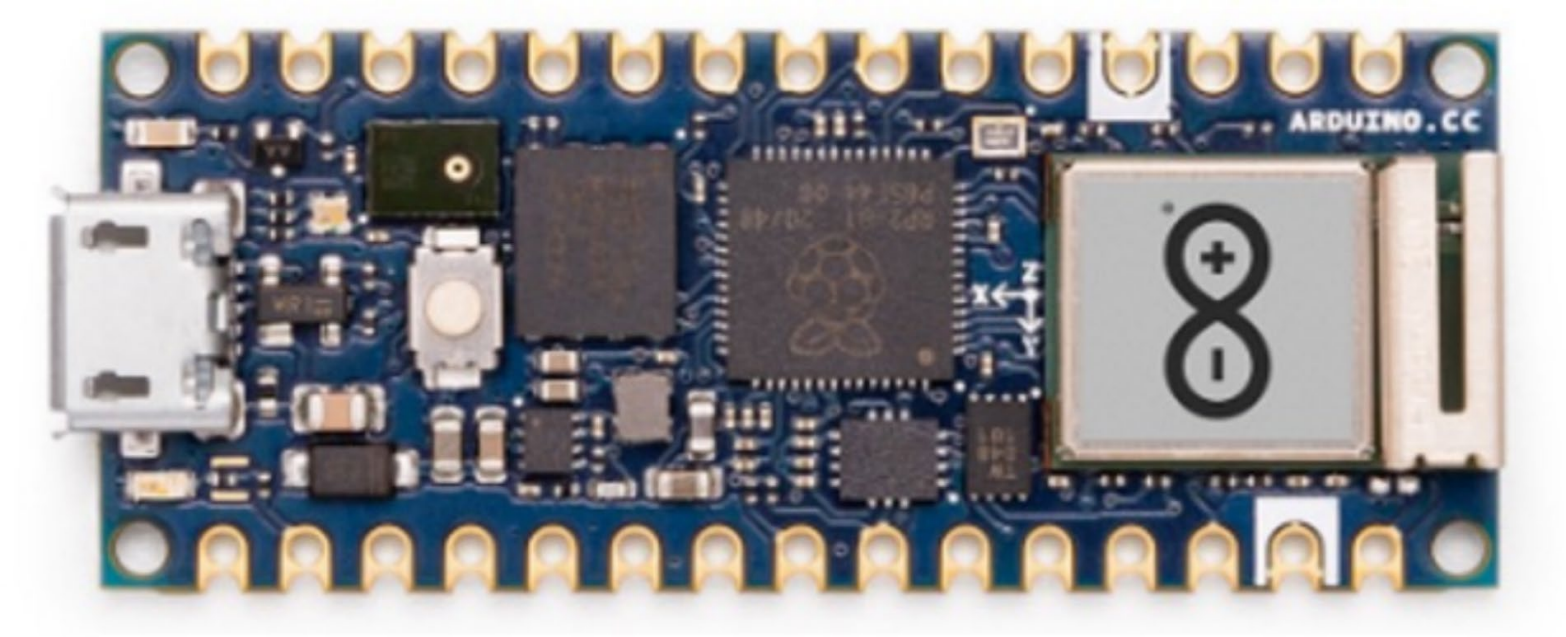
Arduino Nano RP2040.

The Arduino Nano RP2040 Connect device is designed to encapsulate the Raspberry PiRP2040 microcontroller in a compact nano-sized form. The device uses both Bluetooth^®^ and WiFi connectivity and possesses an accelerometer and gyroscope. In addition, it incorporates artificial intelligence technologies. Figure 5 displays the Arduino Nano RP2040 type utilized to develop the proposed system.

#### 3.1.2 IMU

**Figure 6:**
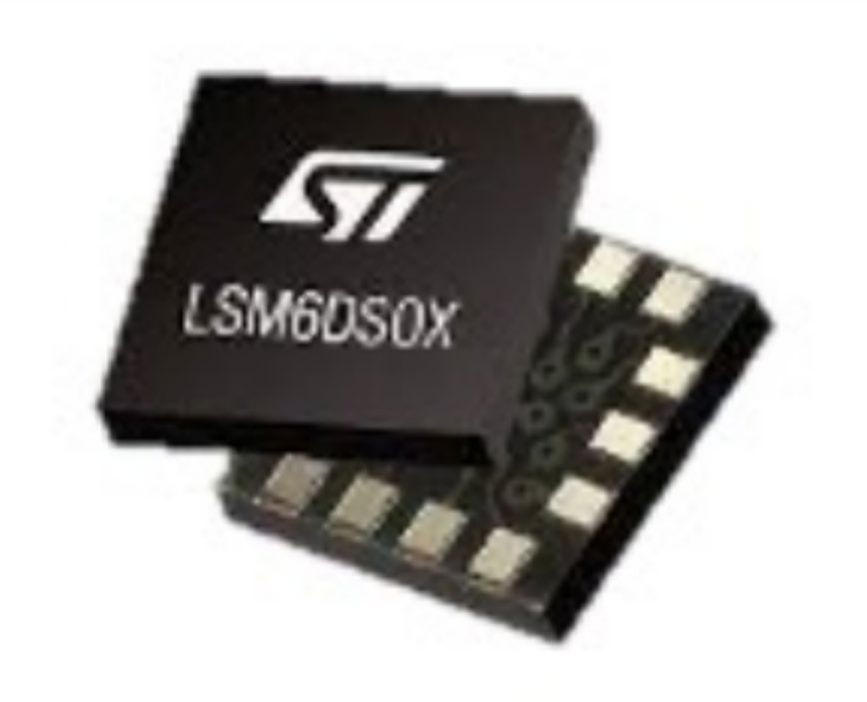
Inertial measurement unit.

IMUs are primarily employed in various devices for measuring velocity, orientation, and gravitational force. In its prior technological iteration, an IMU comprises two sensor types: accelerometers and gyroscopes. Accelerometers are utilized to quantify inertial acceleration, whereas gyroscopes measure angular rotation. Typically, both sensors provide three degrees of freedom to measure along three axes. Capacitive accelerometers, the most frequently used type, rely on changes in electrical capacitance to determine acceleration. When subjected to acceleration, the distance between the capacitor plates within the sensor changes as the diaphragm moves. Within the IMU, the gyroscope quantifies instantaneous angular velocity, typically expressed in units of degrees per second. The IMU device that is utilized in the framework of the proposed system is presented in Figure 6.

#### 3.1.3 Barometer

**Figure 7:**
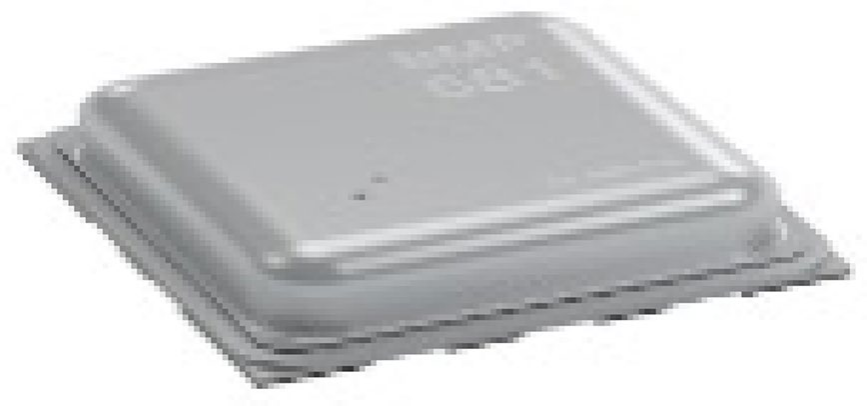
Barometer device.

Barometers are highly responsive devices employed to measure atmospheric pressure at a given location, in which the fluctuations in air pressure at varying altitudes are employed to determine the changes in elevation at specified points. The ability of an IMU to precisely assess changes in height is susceptible to the influence of weight. Therefore, using a barometer facilitates quantifying vertical displacement within the system. The barometer device used in the proposed system is depicted in Figure 7.

#### 3.1.4 Temperature sensor (MAX30205) device

**Figure 8:**
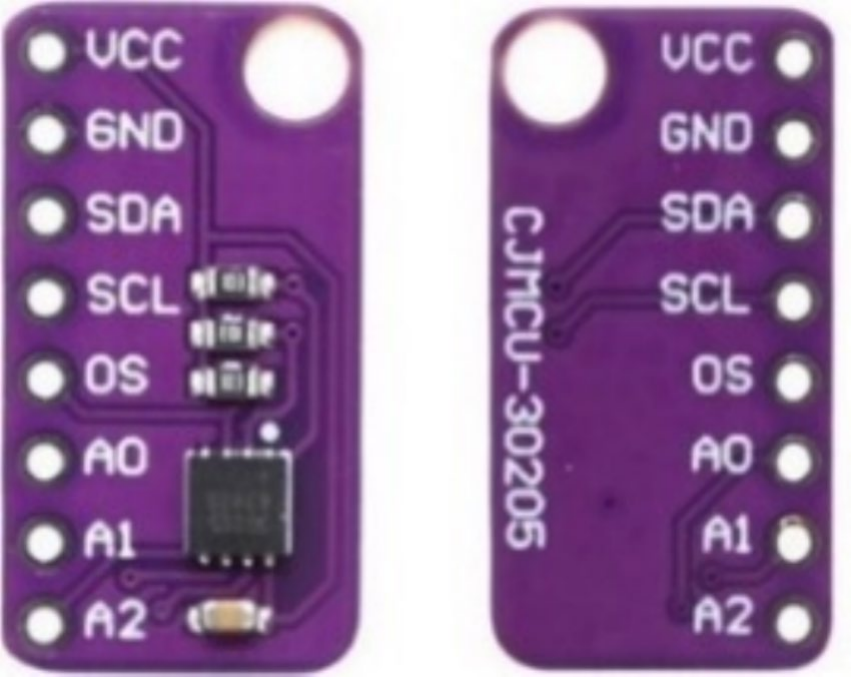
MAX30205 device.

MAX30205 employs a negative temperature coefficient thermistor to measure the temperature by detecting variations in resistance in response to temperature fluctuations. This thermistor is placed in direct contact with the target object, typically the skin, and its resistance is measured by passing a small current through it and recording the resultant reduction in voltage. In addition, the sensor incorporates a digital filter and integrator to process the thermistor output, yielding a high-resolution digital representation of the measured temperature. The digital filter and integrator employ oversampling and noise-shaping techniques to enhance the precision and resolution of the temperature measurement. The MAX30205 sensor used to measure the temperature in the proposed system is depicted in Figure 8.

#### 3.1.5 Oximeter pulse sensor device

**Figure 9:**
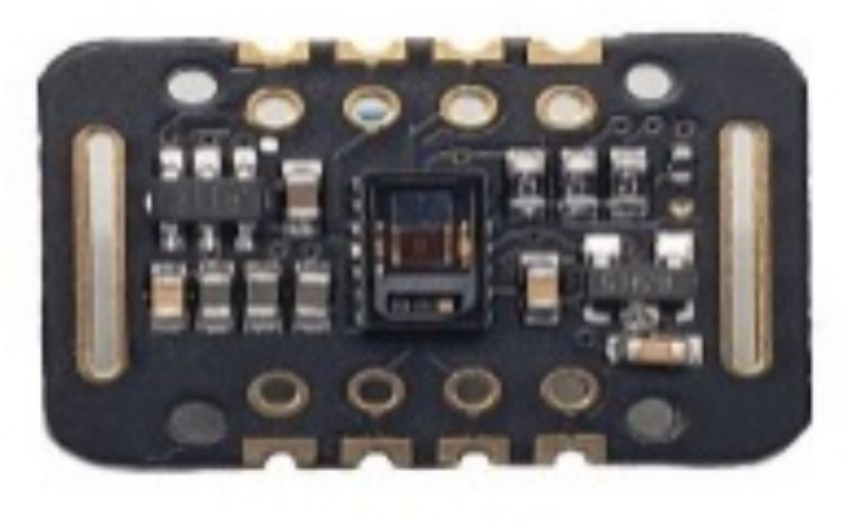
Sensor (MAX30102).

The oximeter pulse sensor operates on photoplethysmography (PPG) principles, a volumetric measurement technique achieved through optical means. PPG quantifies oxygen volume by analyzing variations in light absorption within the body. The device aids in monitoring respiratory levels and various circulatory parameters in the blood. In addition, it enables the calculation of heart rate based on peaks detected in the signal (Yang et al., 2017). Figure 9 illustrates the oximeter pulse sensor used in the proposed system.

### 3.2 Datasets

#### 3.2.1 FallAllD: movement pattern detection standard data

FallAllD constitutes a comprehensive open dataset that encompasses human falls and activities of daily living, as simulated by 15 participants (Saleh et al., 2021). The dataset comprises 26,420 files, collected via three data loggers worn on the users’ waist, wrist, and neck. The motion signals were captured using an accelerometer (Acc), gyroscope (Gyr), and barometer (Bar); the magnetometer was excluded from this study. These sensors were efficiently configured to align with potential applications such as fall detection, prevention, and HAR (Saleh et al., 2021). Table 1 lists the features of the dataset.

**Table 1:** Features of the standard FallAllD dataset.

Feature name	Data type	Feature type	Description	Sensor
Acc X	Float	Numerical, Continuous	X-axis acceleration	Accelerometer
Acc Y	Float	Numerical, Continuous	Y-axis acceleration	Accelerometer
Acc Z	Float	Numerical, Continuous	Z-axis acceleration	Accelerometer
Gyr X	Float	Numerical, Continuous	X-axis rotational speed	Gyroscope
Gyr Y	Float	Numerical, Continuous	Y-axis rotational speed	Gyroscope
Gyr Z	Float	Numerical, Continuous	Z-axis rotational speed	Gyroscope
RSSI	Integer	Numerical, Continuous	Received signal strength indicator (RSSI) (Wi-Fi/Bluetooth signal power)	Wireless Communication

#### 3.2.2 Custom collected data: localization

A total of 260 samples were collected from the three sensor types: Acc, Gyr, and a received signal strength indicator (RSSI). Every sample included a sequence of sensor readings with corresponding timestamps. In addition, the collection consisted of 111 examples linked to specific locations, as localization depended on these labeled instances as a definitive data source. The features of the customized dataset collected from the proposed framework are listed in Table 2.

**Table 2:** Features of the customized FallAllD dataset.

Feature name	Data type	Feature type	Description	Sensor
Acc X	Float	Numerical, Continuous	X-axis acceleration	Accelerometer
Acc Y	Float	Numerical, Continuous	Y-axis acceleration	Accelerometer
Acc Z	Float	Numerical, Continuous	Z-axis acceleration	Accelerometer
Gyr X	Float	Numerical, Continuous	X-axis rotational speed	Gyroscope
Gyr Y	Float	Numerical, Continuous	Y-axis rotational speed	Gyroscope
Gyr Z	Float	Numerical, Continuous	Z-axis rotational speed	Gyroscope
Bar Pressure	Float	Numerical, Continuous	Atmospheric pressure	Barometer
Bar Temperature	Float	Numerical, Continuous	Temperature	Barometer

### 3.3 Preprocessing

**Figure 10:**
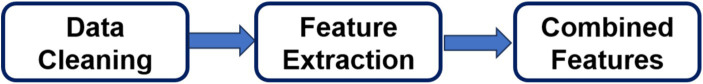
Preprocessing steps.

This section comprehensively explains the feature engineering and preprocessing procedures employed in the current methodology, emphasizing the transformation of raw sensor data into meaningful and actionable features. Figure 10 displays the preprocessing approach for enhancing the proposed system. The data from the various sensors was processed to account for differing data shapes and sensor-specific characteristics:

1. **Data cleaning:** The raw sensor data was first cleaned by converting string representations of lists into actual lists using the ast.literal_eval function.
2. **Feature extraction:** Statistical features (mean, standard deviation, and range) were obtained from the accelerometer, gyroscope, and barometer data. This was done using separate functions for each sensor type:

- calculate_features: Used for accelerometer and gyroscope data, which have X, Y, and Z axes.
- calculate_features_rssi: Used for barometer data, which has pressure and temperature readings.

1. **Combined features:** The extracted features from all three sensors were then combined into a single feature array for each sample. This allowed the data to be input for the ML algorithms.

The preprocessing did differ slightly between sensor types due to the different data shapes and characteristics:

- **Accelerometer and gyroscope:** These sensors have three axes (X, Y, and Z), so the calculate_features function calculated the mean, standard deviation, and range for each axis.
- **Barometer:** This sensor has two readings (pressure and temperature), so the calculate_features_rssi function calculated the mean, standard deviation, and range for each reading.

However, the overall preprocessing approach was similar for all sensor types, involving data cleaning and feature extraction to prepare the data for analysis by the ML models.

#### 3.3.1 Data cleaning

The ast.literal_eval function is employed to convert textual representations of lists in the “Acc,” “Gyr,” and “RSSI” columns back into actual lists. The calculate_features function is defined and implemented to obtain statistical features (mean, standard deviation, and range) from accelerometer and gyroscope data. In addition, the calculate_features_rssi function is defined and applied to extract the same statistical features from the RSSI data.

#### 3.3.2 Feature extraction approach

The calculate_features function is created, which derives statistical features from the sensor data, including the mean, standard deviation, and range for each axis (X, Y, and Z for Acc and Gyr; X and Y for Bar). This function is used on the preprocessed Acc, Gyr, and Bar data to derive their characteristics.

#### 3.3.3 Combined features

The retrieved features from the Acc, Gyr, and Bar data are combined into one feature array to be used as an input for ML algorithms.

### 3.4 Classification algorithms

#### 3.4.1 SVM

SVM is a widely used supervised learning method that can be applied to classification and regression tasks. Primarily, it is utilized for classification tasks in the field of ML. The objective of the SVM method is to establish an optimal line or decision boundary that can divide an *n*-dimensional space into various classes, enabling accurate categorization of incoming data points in the future. The optimal decision boundary is referred to as a hyperplane. SVM aims to identify a hyperplane with the largest margin, namely, the greatest distance between data points from different classes. Increasing the margin distance enhances the classification confidence of subsequent data points.

A standard SVM classifier using the one-*vs*-rest methodology was employed as a baseline for evaluating the performance of the proposed late-fusion CNN model in HAR. The SVM was selected as the baseline due to specific considerations.

- **SVM is a well-established** and commonly utilized ML technique for classification tasks, such as HAR. Its performance characteristics are widely recognized, making it a suitable benchmark for assessing new techniques.
- **SVM is easier** to develop and understand than more complex DL models such as CNNs. This facilitates comprehension of the factors contributing to performance disparities between the two strategies.
- **The one-*vs*-rest methodology** is a popular technique for modifying binary classifiers such as SVM for multiclass tasks like HAR. This enables a balanced comparison between SVM and the late-fusion CNN model, both intended for multiclass classification.

#### 3.4.2 Random forest tree

Random forest tree (RFT) is an ML method utilized to address regression and classification tasks. It employs ensemble learning, which integrates multiple classifiers to address intricate issues. The RFT algorithm comprises several decision trees. The “forest” created by the RF algorithm is trained using bagging or bootstrap aggregating. Bagging is an ensemble meta-algorithm that enhances the precision of ML methods. The RF algorithm builds an ensemble of decision trees, typically created via a method called “bagging” or “bootstrap aggregating.” This process involves creating numerous subsets of the original dataset (with the potential for duplication) and training a decision tree on each subset. Each tree in the forest is built using a bootstrap sample, where a sample is selected from the training set with replacement. In addition, when a node is divided during the tree construction, the selected split is no longer the most optimal among all the features. Instead, the selected split is the most efficient among a randomly selected subset of the attributes. Utilizing random subsets for training, encompassing both samples and characteristics, ensures that the trees within the forest are uncorrelated. By utilizing a forest model instead of individual decision trees, the resilience and accuracy of the model are improved.

#### 3.4.3 K-nearest neighbors algorithm

The K-nearest neighbors (K-NN) method categorizes new cases by comparing their resemblance to existing cases and placing the former in the most similar category. The K-NN algorithm retains all the existing data and categorizes a new point by assessing its similarity. When fresh data are introduced, they can be efficiently categorized into a suitable group by utilizing the K-NN method. First, the value K is chosen for the neighbors. Then, the Euclidean distance of K neighbors is computed. The K-nearest neighbors are selected based on the computed Euclidean distance. K-NN functions by determining the data points in the training set closest to the new point requiring classification. The letter “K” in K-NN represents the nearest neighbors to consider. For example, when the value of K is set to 5, the algorithm looks for the five nearest neighbors of the new data point. Once the nearest neighbors are identified, the algorithm performs a majority vote for categorization purposes, allocating the new point to the class most frequently observed among its neighboring points. When performing regression tasks, it is feasible to determine the mean or median of the adjacent data points. The word “nearest” commonly refers to calculating the distances among locations utilizing metrics such as Euclidean, Manhattan, or Hamming distances.

#### 3.4.4 CNNs

The studies were conducted utilizing two distinct datasets: the FallAllD dataset and a custom dataset. The FallAllD dataset, referred to as the standard dataset, was primarily used for HAR. When developing the CNN models for HAR, the raw sensor readings from the Acc, Gyr, and Bar were used directly without feature extraction. In contrast, when using the FallAllD dataset for SVM comparison, feature extraction was performed. The custom dataset was specifically used for localization tasks, where feature extraction was also applied for traditional machine learning algorithms.

The adapted CNNs were created to capture the distinct characteristics of each sensor type:

**Accelerometers** measure acceleration to detect changes in speed and direction. The CNN model for accelerometers was developed to detect variations crucial for recognizing actions such as walking, running, and falling.
**Gyroscopes** measure angular velocity to detect rotational movements. The CNN model for gyroscopes was created to detect rotational movements, which is crucial for recognizing actions such as turning and twisting.
**Barometers** measure air pressure to detect variations in height. The CNN model for barometers was created to detect variations in height, which is crucial for recognizing actions such as ascending stairs or descending.

Each CNN model was specifically constructed to efficiently process the specific data shapes and characteristics associated with its respective sensors. The primary goal of these models was to accurately classify a broad spectrum of human activities. A window of 13 s instead of 20 s was selected for several reasons. An excessively long sliding window is at risk of encompassing extraneous behaviors, potentially confusing the classifier. In contrast, an extremely short window can fail to adequately capture all stages of falls. The suggested duration of 13 s is optimal, as it offers a reasonable timeframe for capturing all stages of falls and HAR activities (Zhang et al., 2019).

The design of the CNN models was impacted by these characteristics in multiple ways:

- **The input shape** of each CNN model was designed to correspond with the data shape of the specific sensor it was built for. The input shape of the accelerometer CNN model was (2,899, 260, 3), representing 2,899 samples of 260 time steps with three axes (x, y, and z).
- **The filter size** of each CNN model was selected to capture the pertinent properties of its corresponding sensor. The filter size of the accelerometer CNN model was selected to capture the brief alterations in acceleration typical of human motion.
- **The number of layers** in each CNN model was selected to strike a compromise between the model’s complexity and its capacity to learn the pertinent information. The accelerometer CNN model featured fewer layers than the gyroscope CNN model due to the simpler nature of the accelerometer data.

**Figure 11:**
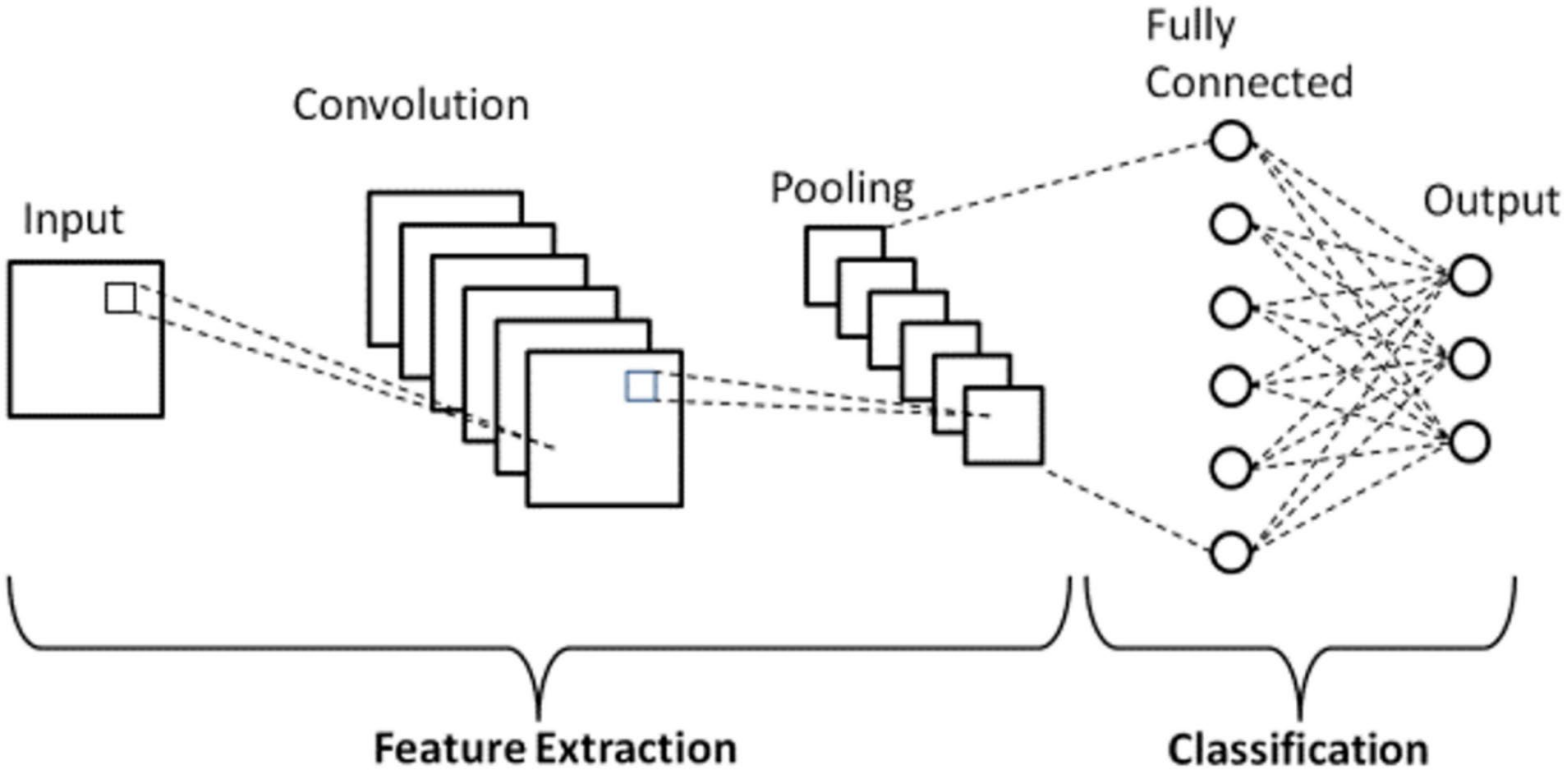
Structure of the CNN.

The data initially obtained from the Acc and Gyr sensors exhibited a sampling frequency of 238 Hz. A deliberate decision was made to reduce the sampling frequency of these sensors to 20 Hz to enhance the ability of the system to detect temporal variations and facilitate a more detailed feature analysis. This adjustment extended the duration of each sensor measurement by an equivalent of approximately 13 s of recorded data. Hence, the dimensions of the Acc and Gyr data matrices transformed to (2,899, 260, 3), with each of the 260 samples representing a duration of 13 s at a sampling rate of 20 Hz. This modification of the CNNs facilitated their ability to analyze sequences of sensor data over a longer timeframe, yielding an enhanced capacity to detect nuanced activity patterns. The Bar sensor was designed to measure the barometric pressure and temperature, acquiring data at a sampling frequency of 10 Hz. Hence, each recorded sensor reading corresponded to the selected time window of 13 s. This produced a modified data matrix with dimensions (2,899, 130, 2). In this case, the 130 samples represented a duration of 13 s at a frequency of 10 Hz, creating 130 samples. This adjustment enabled the CNNs to focus on variations in barometric pressure and temperature within the specified timeframe. This study ensured that the CNN models can efficiently process and extract significant information from the sensor readings by employing this method to modify the sensor data. This conversion was essential for data preprocessing, enhancing the effectiveness of the HAR system. Figure 11 presents the structure of the CNN model.

### 3.5 Late fusion technique

To capitalize on the strengths of different sensor modalities, namely the Acc, Gyr, and Bar, a late-fusion approach was employed in our CNN models. Each sensor type was assigned a dedicated CNN model specifically trained to capture the unique data characteristics pertinent to that sensor. The Acc model was designed to detect linear motion, the Gyr model focused on capturing rotational movements, and the Bar model aimed to identify changes in altitude. These models generated predictions in the form of class probabilities, reflecting the likelihood of each activity.

In the late-fusion approach, we combined these individual predictions using SVM. This process involved aggregating the class probabilities from each sensor-specific CNN model and feeding them into an SVM to form a final, comprehensive prediction. The SVM leveraged the strengths of each model’s predictions, ensuring a robust and accurate classification.

This technique effectively preserved the unique features captured by each sensor, thereby enhancing the overall accuracy and robustness of the HAR system. By integrating predictions at the decision level with the SVM, the late-fusion method provided a more accurate and reliable estimation of human activities compared to single-model approaches. The late-fusion CNN and SVM model demonstrated superior performance in activity classification, thereby validating the efficacy of this multisensory integration strategy.

The choice to utilize late fusion was made due to its various benefits compared to other fusion techniques.

1. **Modularity:** Late fusion enables the separate development and optimization of each sensor-specific CNN model, promoting modularity. The system’s modularity enhances its flexibility and adaptability to various sensor setups or data types.
2. **Interpretability:** Late fusion simplifies the assessment of each sensor’s impact on the final prediction. This is beneficial for comprehending the significance of various sensor modalities for specific activities.
3. **Improved performance:** Late fusion can sometimes enhance performance compared to early fusion, where sensor data is merged before inputting into a single model. Late fusion enables each model to concentrate on extracting features from its unique sensor data, which can be more effective than attempting to learn features from mixed data with diverse properties.

The late-fusion technique was selected for its ability to capitalize on the advantages of several sensor modalities and merge their predictions to create a more precise and resilient HAR system. The late-fusion CNN model’s performance was assessed based on specific metrics and criteria:

- **Validation accuracy:** The precision of the model on a validation subset utilized to assess the model’s performance during training.
- **Final test accuracy:** The precision of the model on a final test set, a subset of the dataset not utilized for training or validation, is employed to evaluate the model’s performance on new data.
- **A classification report** is a detailed analysis of a model’s performance, including precision, recall, and F1-score for each class (i.e., each type of human activity).
- **A confusion matrix** is a tabular representation that displays the counts of true positives, false positives, true negatives, and false negatives for each class.

The metrics and criteria were utilized to evaluate the model’s efficacy in categorizing human actions precisely. The late-fusion CNN model demonstrates good validation and test accuracies and strong performance in the classification report and confusion matrix, indicating its effectiveness for HAR.

### 3.6 Evaluation metrics

Evaluation metrics are essential for evaluating the effectiveness of ML and DL models. Evaluation metrics also assist in choosing models and adjusting hyperparameters. As various jobs necessitate specific measures, using the appropriate metrics is crucial for accurately interpreting model outcomes. In this study, we employed the following evaluation metrics (Equations 1–4):

(1) $$\mathrm{Accuracy}=\frac{TP+TN}{TP+FP+FN+TN}\times 100\%\text{.}$$

(2) $$\mathrm{Recall}=\frac{TP}{TP+FN}\times 100\%\text{.}$$

(3) $$\mathrm{Precision}=\frac{TP}{TP+FP}\times 100\%\text{.}$$

(4) $$\mathrm{Fscore}=\frac{2*\mathrm{preision}*\mathrm{Sensitivity}}{\mathrm{preision}+\mathrm{Sensitivity}}\times 100\%\text{.}$$

Where True Positive (TP) indicates a correct positive prediction; False Positive (FP) indicates an incorrect positive prediction; False Negative (FN) indicates an incorrect negative prediction; and True Negative (TN) indicates a correct negative prediction. These metrics provide a comprehensive understanding of the models’ accuracy, precision, recall, and overall effectiveness in classifying human activities. By employing these metrics, the study ensured robust and reliable performance evaluation, highlighting the strengths and weaknesses of both deep learning and traditional machine learning approaches.

## 4 Experimental results

This section presents the proposed wearable system for human motion sensing technologies.

### 4.1 Experimental setup

Developing a wearable system for human motion sensing technologies requires substantial hardware and software. Tables 3, 4 present these hardware and software requirements, respectively.

**Table 3:** Hardware requirements.

Devices	Type
Arduino^®^ Nano RP2040 Connect	Pull up Resistors (4.7 kΩ)
Barometric pressure sensor (BMP581 Qwiic)	Active buzzer (LTE12-03)
Human body temperature sensor (MAX30205)	Battery (3.7 V)
Pulse sensor & oximeter pulse (MAX30102)	Circuit charger + boost voltage
PCB	3D Model

**Table 4:** Software requirements.

Library	Modules/functions
Pandas	“pd.read_excel,” “pd.sample,” “pd.reset_index,” “pd.DataFrame.apply,” “pd.concat”
Numpy	“np.mean,” “np.std.,” “np.max,” “np.min,” “np.array,” “np.hstack”
Ast	“ast.literal_eval”
Sklearn.model_selection	“train_test_split”
Sklearn.ensemble	“RandomForestClassifier”
Sklearn.metrics	“classification_report,” “accuracy_score”
Sklearn.neighbors	“KNeighborsClassifier”
Sklearn.preprocessing	`StandardScaler`
Sklearn.svm	“SVC”
Sklearn.multiclass	“OneVsRestClassifier”
Keras.models	“load_model”
Keras	“Sequential”
Google.colab	“drive.mount”

### 4.2 Splitting data

Splitting a dataset into two sections enables the assessment of the performance of ML and CNN models, aiding in model selection, hyperparameter tuning, and early halting decisions. Table 5 displays the standard and customized datasets for splitting.

**Table 5:** Splitting the datasets.

Split	Number of samples
Standard dataset	
Training 60%	1739
Validation 20%	579
Test 20%	581
Custom dataset	
Training 60%	66
Validation 20%	22
Test 20%	23

### 4.3 Hyperparameter optimization for all sensors

Achieving optimal performance for CNN models across different sensor types—Acc, Gyr, and Bar—requires a comprehensive hyperparameter optimization strategy. This strategy involves tuning key parameters such as epochs, batch sizes, learning rates, dropout rates, and regularization techniques to enhance model accuracy and robustness.

#### 4.3.1 Accelerometer model

For the accelerometer model, the optimization focused on epochs, batch sizes, and learning rates. Combinations of 10 and 20 epochs, batch sizes of 32 and 64, and learning rates of 0.0001 and 0.001 were evaluated. The optimal configuration, consisting of 20 epochs, a batch size of 64, and a learning rate of 0.001, resulted in a test accuracy of 86.20%. This configuration ensured sufficient training duration and stability while balancing convergence speed and precision.

#### 4.3.2 Gyroscope model

The hyperparameter optimization for the gyroscope model incorporated L2 regularization and a dynamic learning rate schedule. The model utilized 20 epochs, a batch size of 16, and an initial learning rate of 0.0005. A learning rate scheduler was applied to halve the learning rate after 5 epochs, enhancing the model’s fine-tuning capability during later training stages. Additionally, dropout layers with a rate of 0.5 were used to mitigate overfitting. This combination of regularization, dynamic learning rate adjustment, and early stopping produced a robust model capable of effectively interpreting gyroscope data.

#### 4.3.3 Barometer model

The optimization process for the barometer model included a deeper network architecture with multiple convolutional and dense layers, each followed by Leaky ReLU activations and dropout regularization. An initial learning rate of 0.001, which decayed exponentially every 10,000 steps, allowed for gradual refinement of the learning process. The model was trained for up to 50 epochs with a batch size of 32, employing early stopping and model checkpointing to prevent overfitting and to save the best-performing model. This comprehensive approach ensured that the model effectively captured the nuances of barometric data, thereby enhancing its predictive accuracy.

### 4.4 Results of standard data

#### 4.4.1 Results of the CNN model

**Figure 12:**
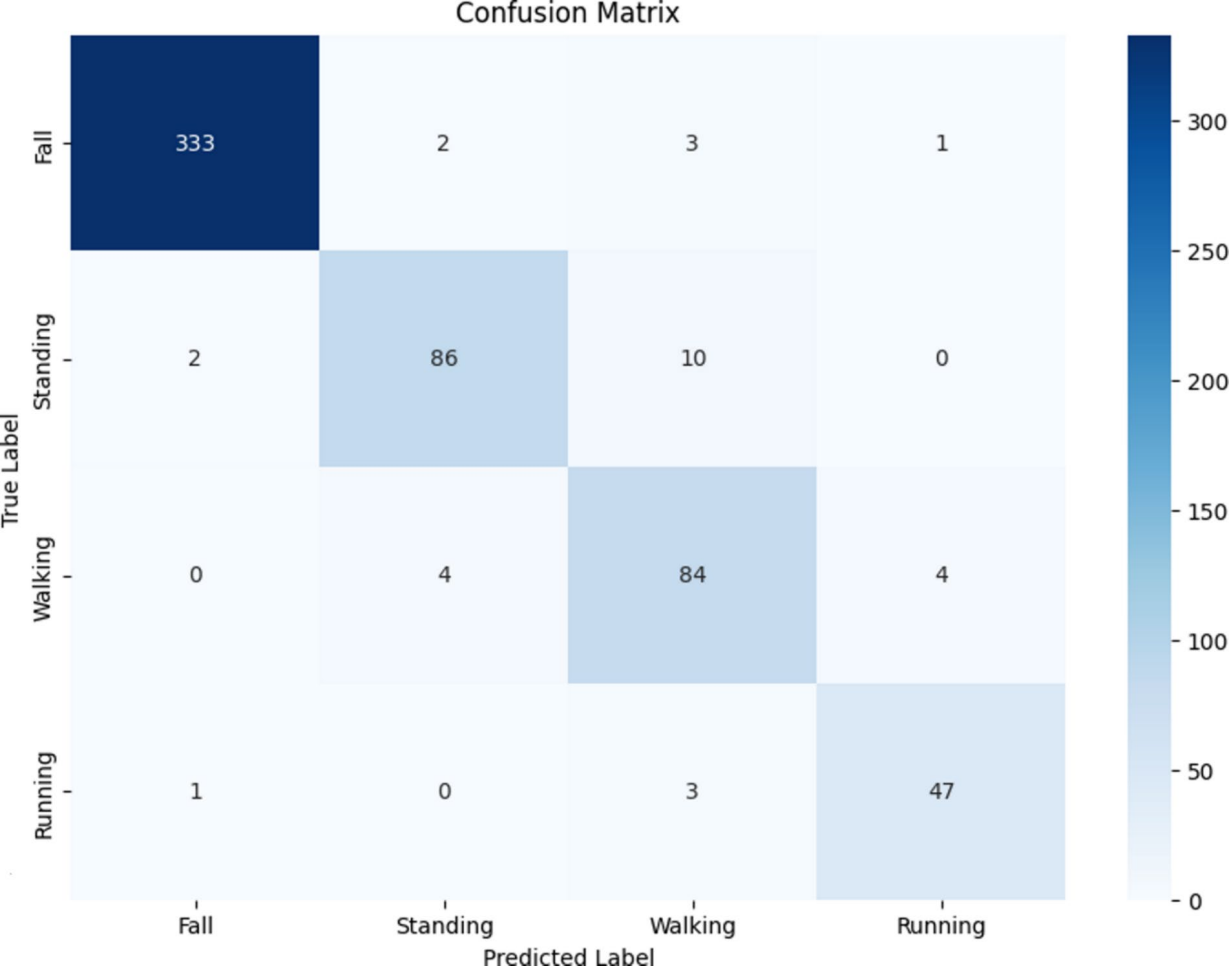
Confusion matrix of the results of the late-fusion CNN-based model.

The results obtained from the late-fusion CNN-based model exhibited remarkable utility, demonstrating its substantial potential to enhance both the accuracy and precision of HAR systems. Throughout the experiments, the late-fusion CNN-based model consistently delivered outstanding performance metrics. The validation accuracy was 98.35%, with a final test accuracy of 94.83%. For a more comprehensive evaluation of the model’s performance, please refer to the classification report in Table 6 and the confusion matrix illustrated in Figure 12. The outcomes presented in this study provide compelling evidence of the effectiveness of the late-fusion CNN model in accurately classifying human activities. The model achieved exceptional accuracy, recall, and F1 scores across various activity classes, emphasizing its ability to accurately distinguish various activities.

**Table 6:** Classification report for the late-fusion CNN model.

	Precision	Recall	F1-score	Support
Fall	0.99	0.98	0.99	339
Standing	0.93	0.88	0.91	98
Walking	0.84	0.91	0.87	92
Running	0.90	0.92	0.91	51
Accuracy			0.95	580
Macro average	0.92	0.92	0.92	580
Weighted average	0.95	0.95	0.95	580

#### 4.4.2 Results of the SVM model

**Figure 13:**
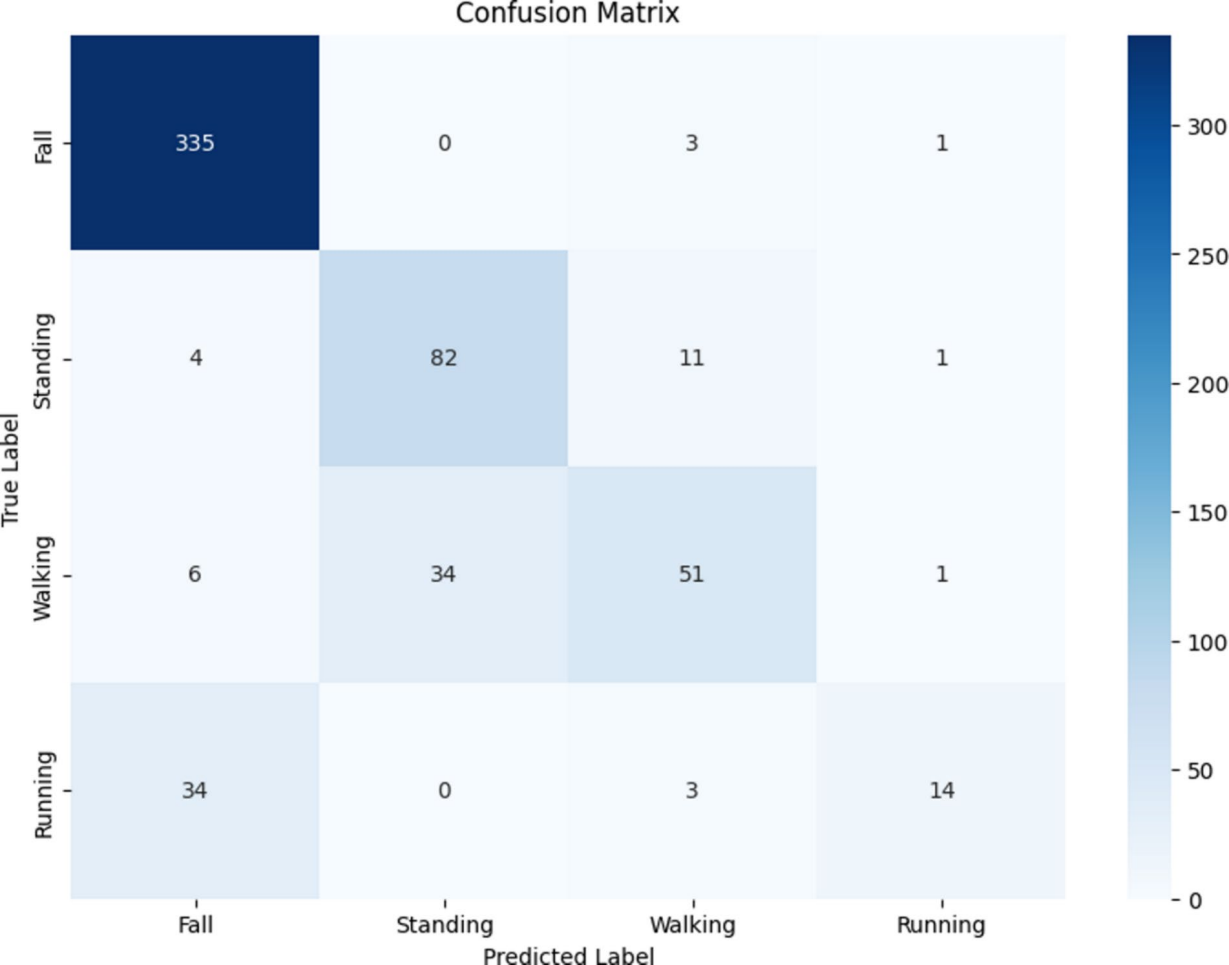
Confusion matrix for the traditional SVM model.

Employing a traditional SVM classifier with a one-*vs*-rest approach led to lower performance metrics, as the validation set accuracy was 87.07%, and test accuracy was 83.10%. The 262 performance details of the SVM model are reported in the classification report in Table 7, and the confusion matrix is displayed in Figure 13. Although the SVM model demonstrates satisfactory performance, it is significantly outperformed by the late-fusion CNN-based model, with the latter achieving higher validation accuracy. This outcome emphasizes the superior ability of the CNN model to accurately classify human activities. The present findings unequivocally establish that the developed CNN model, when combined with the late-fusion technique, substantially enhances the accuracy of HAR. In addition, the classification report provides empirical evidence of its effectiveness in distinguishing a wide range of activities. The impressive accuracy of the CNN model at 95% is somewhat constrained by the limited availability of datasets featuring diverse sensor types and the relatively small dataset size, comprising only 2,899 samples. To further advance HAR systems, future investigations can explore utilizing larger and more diverse datasets to continue improving the accuracy and robustness of these models.

**Table 7:** Classification report for the traditional SVM classifier.

	Precision	Recall	F1-score	Support
Fall	0.88	0.99	0.93	339
Standing	0.71	0.84	0.77	98
Walking	0.75	0.55	0.64	92
Running	0.82	0.27	0.41	51
Accuracy			0.83	580
Macro average	0.79	0.66	0.69	580
Weighted average	0.83	0.83	0.81	580

### 4.5 Results of the custom collected data

#### 4.5.1 Results of the RFT

**Figure 14:**
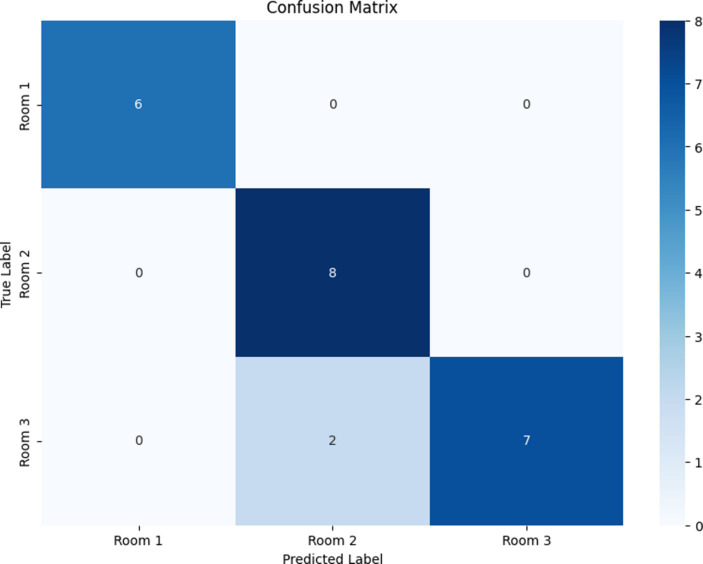
Confusion matrix for the random forest model.

The results provide encouraging possibilities in the realm of indoor localization via the use of ML methodologies. Although the dataset was limited, with only 111 examples, the principal model used (the RF classifier) revealed strong performance in accurately identifying the position of users utilizing sensor data. The model demonstrated a commendable overall accuracy of 91.30%. The classification report offers comprehensive metrics for each class, further clarifying the model’s performance. The metrics of precision, recall and F1-score were calculated for each location class (Room1, Room2, and Room3), as presented in the classification report provided in Table 8. The confusion matrix of the RF model is illustrated in Figure 14.

**Table 8:** Classification report for the random forest model.

	Precision	Recall	F1-score	Support
Room 1	1.0	1.0	1.0	6.0
Room 2	0.8	1.0	0.89	8.0
Room 3	1.0	0.78	0.88	9.0
Accuracy			0.91	23
Macro average	0.93	0.93	0.92	23
Weighted average	0.93	0.91	0.91	23

#### 4.5.2 Results of the K-NN model

**Figure 15:**
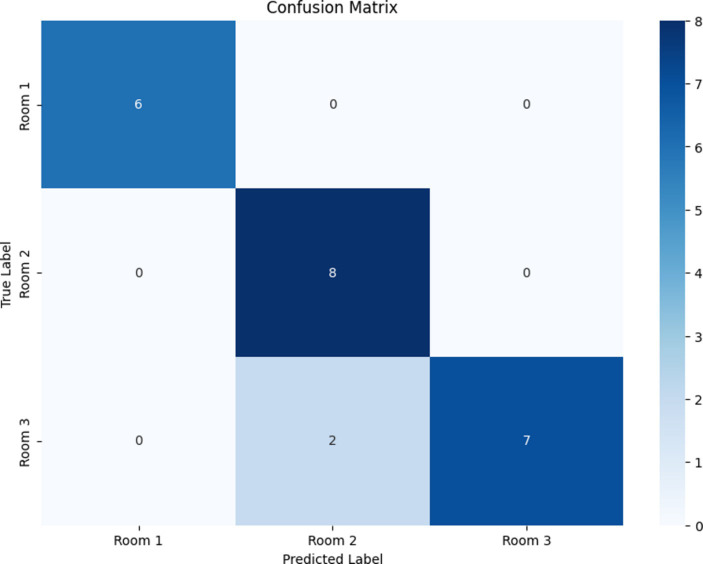
Confusion matrix of the K-NN model.

These metrics provide essential insight into the model’s capacity to accurately categorize each site. The confusion matrix offers a graphical depiction of the model’s predictions compared to the actual ground truth, facilitating the evaluation of true positives, false positives, true negatives, and false negatives. The findings illustrate the resilience of the RF model in indoor localization, indicating its potential for practical applications. With a larger dataset, the model’s performance can improve in robustness and accuracy. The classification report and confusion matrix of the K-NN model are presented in Table 9 and Figure 15, respectively.

**Table 9:** Classification report of the K-NN model.

	Precision	Recall	F1-score	Support
Room 1	0.56	0.83	0.67	6
Room 2	0.71	0.56	0.63	9
Room 3	0.86	0.75	0.80	8
Accuracy			0.70	23
Macro average	0.71	0.71	0.70	23
Weighted average	0.72	0.70	0.70	23

## 5 Discussion

Wearable devices have enabled a range of functions, including recording activities, monitoring wellbeing, and interacting with computers, all aimed at evaluating and improving users’ everyday habits. These applications make use of low-power sensors on mobile and wearable devices to facilitate HAR. The system proposed in this study utilizes CNNs within a late-fusion framework to analyze and integrate data from various sensors for precise HAR specifically designed for healthcare applications. Processing inputs from accelerometers, gyroscopes, and other sensors provide a comprehensive and dynamic representation of patient movements, facilitating accurate and real-time monitoring of physical activities.

The advanced approach to HAR provides significant benefits in the healthcare sector by enabling continuous, non-invasive monitoring of users’ physical activities, contributing to personalized healthcare plans, early detection of potential health issues, and enhanced user care. The proposed system’s high accuracy and reliability in activity recognition can support healthcare professionals in making informed decisions, optimizing treatment plans, and monitoring user recovery processes, improving overall user outcomes.

This study conducted extensive preprocessing to prepare the dataset for training the ML model. The preparation procedures included importing the dataset from an Excel file and performing random shuffling to provide impartial training data. The string representations of the lists in the “Acc,” “Gyr,” and “RSSI” columns were transformed into concrete lists. Then, the sensor data were analyzed to obtain important statistical parameters (e.g., mean, standard deviation, and range) like those for the feature engineering and traditional SVM preprocessing section above. The features were employed as input variables to train the model. The features from “Acc,” “Gyr,” and “RSSI” were merged to form a single array of features for each sample. Table 10 lists the performance of ML and the standard and customized dataset of the CNN model. The study demonstrated that the late-fusion approach utilizing CNNs outperformed traditional HAR methods, with the former achieving a test accuracy of 94.83% compared to that of the SVM classifier at 83.10%. These findings highlighted the effectiveness of using multisensory data through advanced DL techniques, indicating a substantial advancement in accurately classifying diverse human activities. They also emphasized the potential of CNN-based models in setting new standards for HAR applications and the importance of integrating complex sensor data for enhanced performance.

**Table 10:** Test and validation accuracies of the four models.

	Standard data for HAR				Custom data for localization			
	CNN	SVM	Random forest	K-NN	CNN	SVM	Random forest	K-NN
Validation %	98.35	87.07	96.12	92.03	94.44	72.22	89	70
Testing %	95	83	95.52	89.31	82.60	52.17	91	70

This study recommends, in light of the system’s exceptional performance and the accuracy of the sensors used, that future research efforts focus on the following:

1. **Dataset size:** The accumulation of more diverse and extensive datasets. Such endeavors will bolster the system’s robustness across various scenarios and facilitate the exploration of new dimensions in HAR. In addition, the research community is encouraged to explore integrating these refined datasets with the system to enhance its efficacy and applicability in real-world contexts. This collaborative approach promises to set new benchmarks in the field, extending the frontiers of HAR technology.
2. **Sensor fusion challenges:** Combining data from various sensors such as accelerometer, gyroscope, and barometer can be difficult because of differences in sample rates, data formats, and sensor-specific traits. The study addressed this issue by creating specialized CNN models for individual sensor types to capture their distinct characteristics and merge the data successfully in a subsequent phase.
3. **Integration of additional sensors:** Enhancing the process by integrating other sensors like heart rate monitors or electromyography (EMG) sensors can provide a more thorough understanding of human mobility and physiological reactions.
4. **Computational complexity:** CNNs can be costly in terms of the computer resources required for training and deployment. In the future, this issue can be resolved by improving the architecture of the CNN models using methods such as pruning or quantization to decrease the model size or utilizing cloud computing resources for training and inference.
5. **Real-world applicability:** The model’s performance in practical situations can vary from its performance on the test dataset due to differences in sensor placement, user behavior, and ambient variables. It can collect and test the model using a more diverse dataset that accurately reflects real-world scenarios to address this issue.

The applicability of the results in the research to different HAR applications and datasets is contingent on certain factors:

- Activity similarity: The behaviors discussed in the study, such as walking, running, ascending stairs, and falling, are frequently used in various HAR applications. The techniques and models presented in the research can be directly applied or readily adjusted for similar HAR circumstances.
- Sensor configuration: The sensor configuration utilized in the paper, consisting of an accelerometer, gyroscope, and barometer, is frequently employed in various HAR applications. If the sensor setup is substantially different, such as using varied sensor kinds or a variable quantity of sensors, the models can require adjustments or retraining to accommodate the new data.
- Data quality and quantity: The performance of models can be considerably affected by the quality and quantity of data used for training and testing. The FallAllD dataset in the paper was minimal, perhaps restricting the models’ applicability to bigger, more varied datasets. The models’ generalizability can be enhanced by retraining them on a larger and more diverse dataset.
- Variability: The study recognizes that the model’s performance can vary in real-world situations compared to its performance on the test dataset due to differences in sensor placement, user behavior, and ambient variables. Hence, it is crucial to consider these elements when using the methodology in various situations.

## 6 Conclusion

The primary objective of this study is to investigate the capabilities and effectiveness of a multisensory approach, specifically the combination of an IMU and a barometer, to observe and track human movement. The empirical findings support the idea that integrating a triaxial accelerometer, a triaxial gyroscope, and a barometer enhances precision in recognizing various human movement patterns. This enhancement is further reinforced by incorporating an additional filter algorithm, effectively distinguishing between diverse movement patterns, such as standing, falling, running, and walking. In addition, the comprehensive monitoring of various physiological indicators (e.g., cardiovascular rate, sphygmomanometer readings, and thermal body states) provides an additional layer of diagnostic accuracy. This array of capabilities represents a significant advancement in the field of geriatric care, with the potential to mitigate adverse consequences associated with unforeseen movement-related incidents, including falls.

The late-fusion convolutional neural network model in this study improves HAR by achieving a final test accuracy of 94.83%, outperforming the standard SVM classifier using a one-*vs*-rest approach, which had an accuracy of 83.10%. Using customized CNNs for each sensor type and employing the late-fusion strategy to combine their predictions has proven beneficial. The improved precision in HAR, mainly in distinguishing between behaviors such as falling and regular everyday activities, has significant implications for fall detection systems, personalized health monitoring, and sports performance analysis. Future research will focus on improving the methodology using larger and more diverse datasets, adding further sensors, enhancing real-time processing, and introducing explainable AI techniques. This research ultimately seeks to enhance persons’ quality of life by developing more precise and efficient HAR systems that can be integrated into wearable devices.

## Data availability statement

The raw data supporting the conclusions of this article will be made available by the authors, without undue reservation.

## Author contributions

MA: Conceptualization, Investigation, Methodology, Resources, Supervision, Validation, Writing – original draft, Writing – review & editing. AM: Conceptualization, Data curation, Formal analysis, Investigation, Methodology, Resources, Software, Supervision, Validation, Writing – original draft, Writing – review & editing. AAl: Methodology, Supervision, Writing – review & editing. MF: Methodology, Validation, Writing – review & editing. SC: Conceptualization, Investigation, Software, Writing – review & editing. AAf: Data curation, Methodology, Writing – review & editing. AAh: Writing – review & editing. OR: Conceptualization, Investigation, Methodology, Software, Writing – original draft, Data curation. AH: Conceptualization, Investigation, Methodology, Writing – original draft, Writing – review & editing, Software. MT: Writing – review & editing, Conceptualization, Data curation, Investigation, Methodology, Writing – original draft.
